# Partial nail avulsion: habit or evidence based?

**DOI:** 10.1186/1757-1146-4-S1-O46

**Published:** 2011-05-20

**Authors:** Priten Solanki, Peta Craike

**Affiliations:** 1 Podiatry Lecturer, School of Health Sciences, University of Newcastle, Ourimbah, NSW, 2258, Australia; 2 Podiatry Lecturer , School of Health Sciences, University of Newcastle, Ourimbah, NSW, 2258, Australia

## 

Onychocryptosis is a relatively common condition that can cause significant pain and discomfort. Partial nail avulsion (PNA) with phenolisation is a straightforward procedure performed by podiatrists on a daily basis. The procedure has shown a high rate of efficacy and low recurrence rate, and can be performed on high risk patients with close post-operative monitoring and those with concomitant infection. PNA with phenolisation of the nail matrix is a non-invasive procedure that does not require the use of an operating theatre and can be carried out in the podiatrist’s rooms. Pre-operative measures do need to be taken, a local sterile field should be set up and the toe and forefoot should be scrubbed. Once the procedure has been completed healing can be expected in 4-8 weeks. The PNA procedure has been taught to undergraduate podiatrists since the late 1970’s and has developed many small variations in how the procedure is carried out. Variations vary from pre-operative management (type of antisepsis used), phenolisation time (reported to be between 1 to 5 minutes), type of post phenol irrigation (saline, isopropyl alcohol or no irrigation) and post-operative dressing regimen. These variations however are usually operator dependant and based on personal experience. Post-operative management of PNA wounds have attracted a lot of interest, with the use of different dressings (provodone-iodine impregnated gauze and paraffin gauze) and topical medicaments (manuka honey, intrasite gel) aimed at increasing the healing rate and reducing the rate of infection, but quantitative analysis of the colonisation of the wound bed shows a bacterial count of zero after the use of phenol. The case of the phenolised wound is an interesting one, an acute wound that heals by secondary intention and freely discharges for 2-4 weeks postoperatively. After the application phenol and the destruction of all microbiological matter the post-operative focus should be in nurturing the recolonisation of the nail bed. Wounds that have a high as well as a low bacterial count have been shown to have an effect healing rates, but no studies have investigated the microbiological behaviour of a phenolised PNA wound.

